# Molecular Dynamics Simulation of Phosphorylated KID Post-Translational Modification

**DOI:** 10.1371/journal.pone.0006516

**Published:** 2009-08-05

**Authors:** Hai-Feng Chen

**Affiliations:** 1 College of Life Sciences and Biotechnology, Shanghai Jiaotong University, Shanghai, China; 2 Shanghai Center for Bioinformation Technology, Shanghai, China; German Cancer Research Center, Germany

## Abstract

**Background:**

Kinase-inducible domain (KID) as transcriptional activator can stimulate target gene expression in signal transduction by associating with KID interacting domain (KIX). NMR spectra suggest that apo-KID is an unstructured protein. After post-translational modification by phosphorylation, KID undergoes a transition from disordered to well folded protein upon binding to KIX. However, the mechanism of folding coupled to binding is poorly understood.

**Methodology:**

To get an insight into the mechanism, we have performed ten trajectories of explicit-solvent molecular dynamics (MD) for both bound and apo phosphorylated KID (pKID). Ten MD simulations are sufficient to capture the average properties in the protein folding and unfolding.

**Conclusions:**

Room-temperature MD simulations suggest that pKID becomes more rigid and stable upon the KIX-binding. Kinetic analysis of high-temperature MD simulations shows that bound pKID and apo-pKID unfold via a three-state and a two-state process, respectively. Both kinetics and free energy landscape analyses indicate that bound pKID folds in the order of KIX access, initiation of pKID tertiary folding, folding of helix α_B_, folding of helix α_A_, completion of pKID tertiary folding, and finalization of pKID-KIX binding. Our data show that the folding pathways of apo-pKID are different from the bound state: the foldings of helices α_A_ and α_B_ are swapped. Here we also show that Asn139, Asp140 and Leu141 with large Φ-values are key residues in the folding of bound pKID. Our results are in good agreement with NMR experimental observations and provide significant insight into the general mechanisms of binding induced protein folding and other conformational adjustment in post-translational modification.

## Introduction

cAMP response-element binding protein (CREB) as transcriptional activator can stimulate target gene expression in signal transduction upon associating with CREB binding protein (CBP).[1], [2] CREB consists of four domains: C-terminal domain, two hydrophobic glutamine-rich domains (Q1 and Q2), and kinase-inducible domain (KID). [3] After post-translational modification, phosphorylated KID (pKID) can bind the KIX domain of CBP. As a couple of the best characterized transcription factors, the complex has been reported in many researches to reveal relationship between their structures and functions.[3],[4],[5],[6],[7],[8]

**Figure 1:**
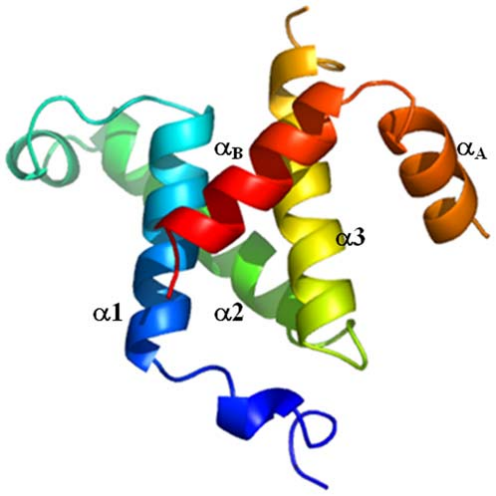
Ribbon representation of NMR structure for pKID-KIX (pdb code: 1KDX). The location of main secondary structures are indicated.

The NMR structure of pKID/KIX complex was released in 1997(pdb code: 1KDX).[7] The complex has five α-helices: α_A_, α_B_, α1, α2, and α3. pKID consists of helix α_A_ from Asp120 to Ser129 and α_B_ from Pro132 to Asp144. Helix α_B_ is almost perpendicular to helix α_A_. KIX includes helices α1 from Gln597 to Ile611, α2 from Arg623 to Tyr640, and α3 from Arg646 to Lys662. The helices α1 and α3 form a shallow hydrophobic groove for the helix α_B_ binding. The helix α_A_ interacts with another face of the helix α3 (see Figure 1).

NMR experiments indicate that apo-pKID is a characteristic unstructured protein. Upon KIX-binding, pKID undergoes a transition from disordered to well folded.[7] This suggests that KIX-binding induces significant conformational change in bound pKID. These experimental observations raise a series of interesting questions. (i) What is the driving force for pKID changing from disordered to well-folded? (ii) What is the difference of the folding pathway between bound and apo-pKID? (iii) Which mechanism does this complex system obey during protein folding? To answer these questions, we utilize all atom molecular dynamics (MD) simulations in explicit solvent to analyze the folding coupled binding[9], [10] in the pKID-KIX complex.

However, all atomic MD simulations are currently restricted to timescale of less than 1µs, which is much shorter than the folding half-time of most proteins at room temperature (at least 1 ms).[11], [12] Fortunately, the unfolding rate increases with temperature, so most proteins unfold in the ns time scale at 498 K.[11] Therefore, MD simulations at high temperature have been widely used[13], [14], [15], [16], [17], [18], [19], [20], [21], [22], [23], [24], [25], [26] to monitor protein unfolding. Furthermore, the experiment confirms the transition state for folding and unfolding is expected to be the same from the principle of microscopic reversibility.[11] Based on these previous works, unfolding simulations at high temperature have also been used in the current study. When we completed this work, a related simulation with Gô model was published about the mechanism of folding and binding for pKID and KIX. [27] At the same time, induced-fit and fly-casting mechanism was given to explain the binding and folding of pKID-KIX complex.[28] Nevertheless, all atomic MD models can provide more detail information about the folding and binding kinetics of the complex.

## Methods

### Room-temperature and high-temperature molecular dynamics simulations

The atomic coordinates of the pKID-KIX complex were obtained from the NMR structure (pdb code: 1KDX).[7] All MD simulations are all-atom explicit solvent and are performed at both 298 K and 498 K. Details of MD protocols are described in elsewhere.[24], [26]

To study the folded state of each solvated system, ten independent trajectories of 10 ns each in the NPT ensemble[29] at 298 K were simulated with PMEMD[30] of AMBER8.[31] To investigate the unfolding pathway of each solvated system, ten independent unfolding trajectories of 20 ns each were performed in the NVT ensemble at 498 K but with the water density at 298 K (i.e. all high-temperature simulations were started from the end of the 10 ns 298 K trajectories). A total of 1 µs trajectories were collected for four systems (bound pKID, apo-pKID, apo-KIX, and apo-KID) at 298 K and for three systems (bound pKID, apo-pKID, apo-KIX) at 498 K, taking about 46,020 CPU hours on the in-house Xeon (1.86 GHz) cluster.

Native contacts for the bound and apo-pKID were monitored to detect the beginning of the unfolded state. It was found that 11 ns at 498 K were needed to reach the equilibrium stage for both bound and apo-pKID, so that the first 11 ns (a total of 110 ns for each system) were used to study the unfolding kinetics and the remaining 9 ns (a total of 90 ns for each system) were used for the unfolded equilibrium state.

### Transition state simulations

According to the definition of transition state (TS), 40 test MD runs for each candidate snapshot were performed to calculate the transition probability (P).[32], [33], [34] TS simulations were done at 498 K to accelerate simulated folding/unfolding rate. The detail methods are described in the previous literature.[24], [26], [35]

### Free energy landscape analysis

The unfolding landscape was determined by calculating normalized probability from histogram analysis.[32] Here we used the fraction of native binding contacts for the helix α_A_(Qb(α_A_)) and for the helix α_B_(Qb(α_B_)) to map the unfolding landscape.

### Data Analysis

Tertiary contact assignment was handled with in-house software.[24] Two non-adjacent residues are in contact when their Cα atoms are closer than 6.5 Å. Secondary structure assignment was performed with DSSP.[36] Representative structures at folding half times were used to construct unfolding pathways. Each representative structure is the closest snapshot to the average of all chosen snapshots at a given half time (within±its standard deviation).

Φ-values were computed with the same strategy to those used in other studies:[33], [37], [38]

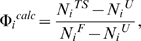
where *N_i_^TS^* is the number of native contacts for residue i at transition state, *N_i_^F^* and *N_i_^U^* is the number of native contacts for residue i at folded and unfolded states.

## Results

### Folded state

**Figure 2:**
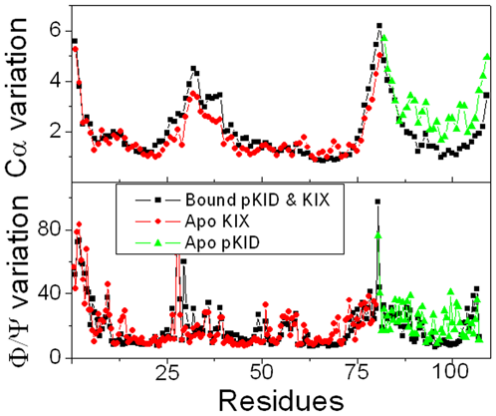
Cα and ψ/Φ variations at folded state for bound and apo-pKID, respectively.

As a reference for the unfolding simulation, 10 trajectories of 10 ns each were simulated at 298 K to analyze the folded state of apo-pKID, apo-KIX, and their complex, respectively. To study the influence of KIX-binding on the stability of bound pKID, Cα and Φ/ψ fluctuations for bound and apo-pKID are illustrated in Figure 2. The Cα variation of bound pKID is significant smaller than that of apo-pKID, especially in the binding domain of the helices α_A_ and α_B_. This suggests that bound pKID becomes less flexible and more stable upon KIX-binding. The Φ/ψ variation of bound pKID is also smaller than that of apo-pKID within the helix α_B_. This suggests that the stability of secondary structure has also improved upon KIX-binding. These results are consistent with the experimental observation that pKID folds into two mutually perpendicular helices from disordered structure upon KIX-binding.[39] Unlike pKID, the variation of tertiary structure for bound KIX is slight larger than that of apo-KIX. Furthermore, the stability of secondary structure for bound KIX does not significantly improve.

**Figure 3:**
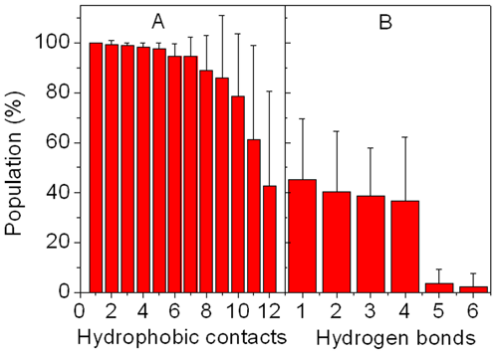
Hydrophobic contacts and hydrogen bonds between pKID and KIX. A: hydrophobic contacts. 1 for Leu138/Ala654, 2 for Tyr134/Ala654, 3 for Ile137/Ile657, 4 for Ile137/Ala654, 5 for Leu141/Ala654, 6 for Leu141/Leu653, 7 for Leu141/Tyr650, 8 for Ala145/Leu599, 9 for Leu128/Tyr658, 10 for Ala145/Tyr650, 11 for Ala145/Leu603, and 12 for Pro146/Leu599. B: hydrogen bonds. 1 for pSer133/Tyr658; 2 for Asp144/Lys606; 3 for Arg131/Tyr658; 4 for Asp140/Lys606; 5 for Arg125/His651; 6 for Leu141/Tyr650.

To study the drive force for conformational adjustment, the interactions between pKID and KIX were analyzed. All possible hydrophobic contacts and hydrogen bonds of the NMR structure were identified with Ligplot [40] and shown in supplementary (Figure S1). The average populations and their standard errors of twelve hydrophobic contacts in ten trajectories are shown in Figure 3A. Twelve stable hydrophobic interactions can be found: Leu138/Ala654, Tyr134/Ala654, Ile137/Ile657, Ile137/Ala654, Leu141/Ala654, Leu141/Leu653, Leu141/Tyr650, Ala145/Leu599, Leu128/Tyr658, Ala145/Tyr650, Ala145/Leu603, and Pro146/Leu599, with population higher than 35%. Surprisingly, the contribution of binding contacts between pKID and KIX is predominated by the helix α_B_, and only a small fraction of native hydrophobic contacts is provided by the helix α_A_(1 out of 12). The tighter binding between helix α_B_ and KIX is consistent with the previous results of mutational experiment and simulation.[3], [27] Besides the hydrophobic interactions, six possible hydrogen bonds were also identified with Ligplot.[40] Figure 3B shows their populations in simulation. The results suggest four stable hydrogen bonds with population higher than 35%. The other two hydrogen bonds are very weak. Notable, there is a stable hydrogen bond between Tyr658 of KIX and phosphorylated Ser133 (pSer133). This is in agreement with the previous result that the phosphate at Ser133 can stabilize α-helix by forming hydrogen bonding interaction.[3], [7], [41] Furthermore, Tyr658 of KIX also contributes one hydrophobic contact to the complex. This suggests that Tyr658 plays a critical role in stabilizing the complex. This is in good agreement with NMR experiment.[7] In summary, KIX-binding introduced more hydrophobic contacts at the interface which are the drive forces for conformational adjustment of bound pKID.

### Unfolding kinetics

**Figure 4:**
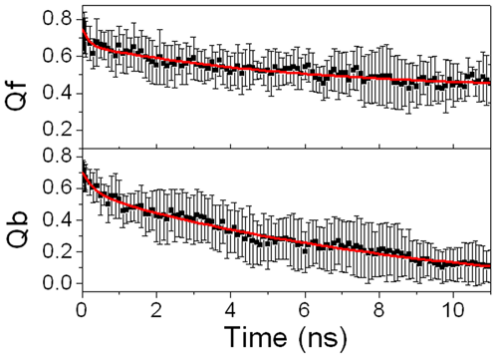
The kinetics fitting for bound pKID.

Native tertiary contacts (Qf) and native binding contacts (Qb) are used to monitor unfolding and unbinding kinetics. Time evolutions of Qf and Qb for bound pKID are shown in Figure 4. Apparently, the tertiary unfolding and unbinding kinetics can be represented well by double exponential functions. This indicates that the tertiary unfolding and unbinding process obeys second order kinetics in the NVT ensemble at high temperature. The fitted kinetics parameters are listed in Table 1. Our kinetics analysis shows that the first unbinding half-time is 0.35±0.13 ns, and the second unbinding half-time is 9.44±1.61 ns. For the tertiary unfolding, the first half-time is 0.17±0.091 ns and the second half-time is 5.98±1.24 ns. This indicates that the tertiary unfolding is much faster than the unbinding, that is, the unbinding of pKID depends on the tertiary unfolding. This is consistent with the result of Gô model. [27] The time evolution of Qf for apo-pKID is shown in supplementary (Figure S2). In contrast, it is found that the tertiary unfolding of apo-pKID obeys first order kinetics, with a half-time of 1.00±0.13 ns, which is obvious faster than the second half-time of the tertiary unfolding for bound pKID. This suggests that the binding of KIX significantly postpones the tertiary unfolding of pKID. This is in agreement with the experimental observations.[7], [8]

**Table 1:** Unfolding kinetics constants for bound pKID.

	τ_1_(ns)	τ_2_(ns)	A_1_	A_2_	B	R^2^
Qb	0.35±0.13	9.44±1.61	0.12±0.018	0.67±0.050	−0.10±0.062	0.98
Qf	0.17±0.091	5.98±1.24	0.086±0.015	0.24±0.018	0.42±0.021	0.91

The curves are fitted by A_1_ *exp*(−t/τ_1_)+A_2_ *exp*(−t/τ_2_)+B.

**Figure 5:**
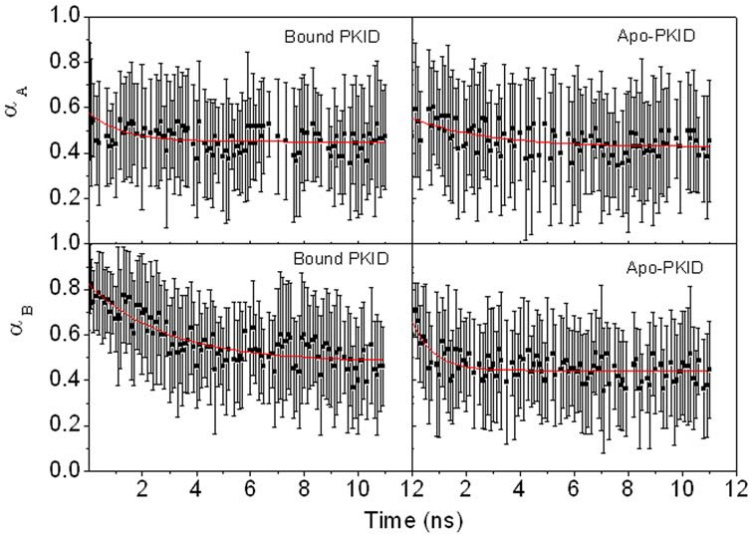
The unfolding kinetics of two helices.

Unfolding kinetics of two helices is also analyzed and presented in Figure 5. The fitted kinetics data are listed in Table 2. Our analysis shows that the helical unfolding obeys first order kinetics under the high-temperature simulation condition. The unfolding half time is 1.24±0.44 ns for the helix α_A_ and 2.86±0.41 ns for the helix α_B_, respectively, in bound pKID. The unfolding half time is 2.73±1.18 ns for the helix α_A_ and 0.77±0.20 ns for the helix α_B_, respectively, in apo-pKID. Surprisingly, the helical unfolding of bound pKID is faster than the tertiary unfolding and unbinding. This is different from other unfolding simulations of helical proteins, for example chymotrypsin inhibitor 2, MDM2 and PAZ.[24], [26], [42] Note also that the unfolding half time of the helix α_B_ for bound pKID are larger than that of apo-pKID, suggesting that KIX-binding stabilizes the helix α_B_ in bound pKID.[43] Furthermore, the unfolding order of helices α_A_ and α_B_ is swapped upon KIX-binding. This suggests that KIX-binding significantly changes the pathway of helical folding for pKID and consistent with the result of p53/MDM2 complex.[24].

**Table 2:** Unfolding kinetics constants.

		τ (ns)	A	B	R^2^
Bound pKID	α_A_	1.24±0.44	0.13±0.020	0.45±0.0072	0.32
	α_B_	2.86±0.41	0.34±0.017	0.48±0.013	0.78
Apo-pKID	Qf	1.00±0.13	0.19±0.015	0.45±0.0031	0.69
	α_A_	2.73±1.18	0.12±0.021	0.43±0.013	0.30
	α_B_	0.77±0.20	0.21±0.032	0.44±0.0060	0.41

The curves are fitted by A*exp*(−t/τ)+B.

### Unfolding landscapes

**Figure 6:**
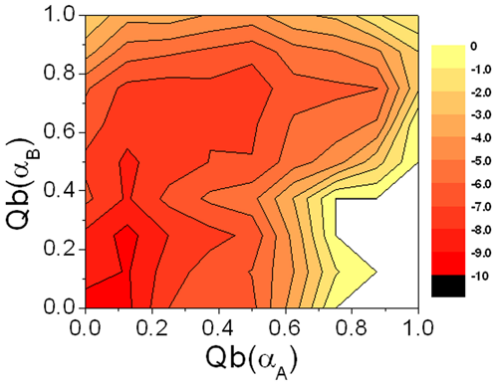
Unfolding landscapes with respect to Qb(αA) and Qb(αB) for bound pKID.

To explore the unbinding order for the helices α_A_ and α_B_, the unfolding landscape of bound pKID with the variables of Qb(α_A_) and Qb(α_B_) is shown in Figure 6. The unfolding landscape shows that the unbinding of the helix α_A_ is happened first while the helix α_B_ is held stable, then is followed by the unbinding of the helix α_B_. This is in agreement with the results that KIX forms tighter interaction with the helix α_B_ than the helix α_A_ in bound pKID.[3]

### Transition state and intermediate state

Kinetics analysis shows that the tertiary unfolding of bound pKID obeys second order kinetics. This suggests that bound pKID unfolds via a three-state process. Therefore, there are two transition states corresponding to two free energy maximums along their unfolding pathways. Between two transition states, there is an intermediate state corresponding to the free energy minimum. Interestingly, NMR relaxation dispersion experiments confirm a single binding intermediate.[8] According to the definition of transition state ensemble (TSE), we have scanned TSE structures from MD snapshots in all 10 high-temperature trajectories for bound pKID.[32] The transition probability curves are further fitted by the Boltzmann equation and shown in supplementary (Figure S3). Our analysis yields 567 snapshots for the first transition state (TS1) and 245 snapshots for the second transition state (TS2). Between two transition states, we capture the intermediate state ensemble. For apo-pKID, the unfolding kinetics suggests that the tertiary unfolding obeys first order kinetics. Therefore, apo-pKID unfolds via a two-state process. This suggests a transition state during the unfolding of apo-pKID. Similar process was performed to scan the transition state for apo-pKID and 144 snapshots for the transition state were found.

The average structures of TS1 and TS2 for bound pKID and TS for apo-PKID are shown in supplementary (Figure S4). For the representative average structure, 78.0% native hydrophobic contacts and 58.1% native binding contacts for the TS1, and 65.9% native hydrophobic contacts and 12.9% native binding contacts for the TS2 are remaining. Apparently, it can be concluded that the TS1 of bound pKID is more native-like than the TS2. For the TS of apo-pKID, 57.1% native hydrophobic contacts are remaining. The structure of intermediate state is shown in supplement (Figure S5). For the intermediate state, the native helical content of α_A_ (about 63.6%) is larger than that of α_B_ (about 53.8%). Besides, there are seven non-specific binding contacts between KIX and the helix α_B_. These interactions can stabilize the final bound state. This is in agreement with the results of NMR experiment. [8]

### Φ-value prediction

**Figure 7:**
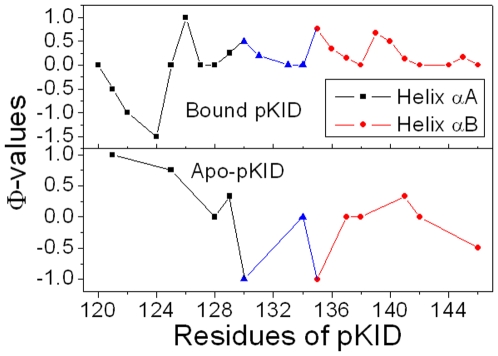
Predicted Φ-values of bound and apo-pKID.

All TSE snapshots were used to predict Φ-values for bound and apo-pKID. Their Φ-values are shown in Figure 7. Φ-values have been widely used for determining key residues in the protein folding by theoretical and experimental investigations.[44], [45], [46], [47] In general, predicted Φ-values of the helix α_A_ are larger than those of the helix α_B_ for apo-pKID. This suggests that the helix α_A_ is more native-like than the helix α_B_ at the apo state. However, the Φ-values of the helix α_B_ are significant larger than those of the helix α_A_ (except Glu126) for bound pKID. This suggests that the helix α_B_ of bound pKID is more native-like than the helix α_A_ upon KIX-binding and consistent with the unfolding kinetics and landscape analysis. Note also that the highest Φ values are found for Asn139, Asp140 and Leu141, suggesting these residues play key role in the folding of bound pKID. A critical role of Leu141, which deeply extends into the hydrophobic groove of KIX, forms three hydrophobic contacts with KIX. This is consistent with the results of Gô model.[27] These predicted Φ values can be confirmed by experiment.

## Discussion

### Comparison with experiment

The structural analysis suggests that the phosphorylated Ser133 (pSer133) of pKID and Tyr658 of KIX are critical residues in stabilizing the complex.[7] Our room temperature simulation illustrates two stable hydrogen bond interactions for pSer133/Tyr658 and Arg131/Tyr658. Besides hydrogen bond, there is also one stable hydrophobic contact between Tyr658 and Leu128. This is in agreement with the mutational experiment that Tyr658Phe decreases 3- to 4-fold binding affinity and Tyr658Ala completely abrogates complex formation.[7]

Furthermore, it has been observed in NMR experiment that the phosphorylation of apo-KID does not lead to a discernible increase in helical content.[7] In order to compare the influence of phosphorylation on helical content, the structures of the last 5 ns are used to calculate the native helical contents of the helices α_A_ and α_B_ for apo-KID and apo-pKID, respectively. The native helical content is 44.5±6.0% for α_A_, and 58.5±4.4% for α_B_ in apo-KID, respectively. The content of native helix is 43.6±7.3% for α_A_, and 53.1±4.9% for α_B_ in apo-pKID, respectively. Within experimental error, the helical content of apo-pKID does not significantly increase upon the phosphorylation. This is in agreement with the observations of NMR experiment.[7]

Finally, we predict Φ-values of pKID (shown in Figure 7) and find that the Φ-values of Asn139, Asp140 and Leu141 are higher than those of other residues for bound pKID. These results are also consistent with the previous report [27] and can be conformed by experiment.

### Convergence and Sampling

**Figure 8:**
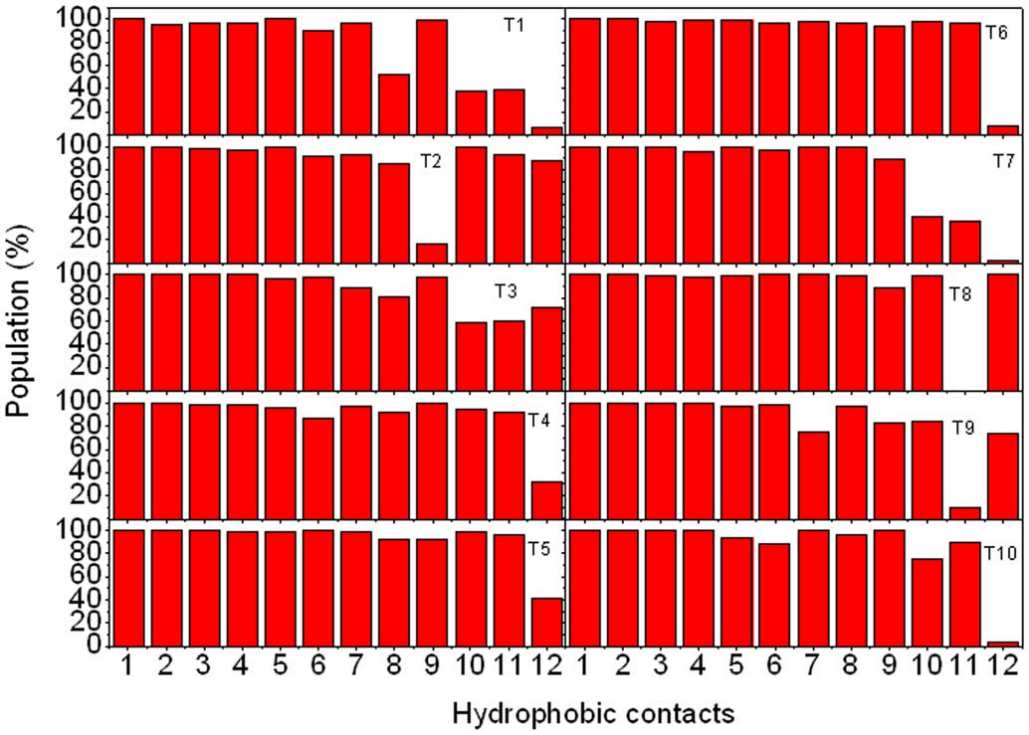
The hydrophobic contacts between pKID and KIX in ten trajectories. (The order of hydrophobic contacts is same to Figure 3).

Ten trajectories were simulated for bound, apo-pKID and KIX, respectively. Firstly, we want to know if multiple trajectories are necessary to this study. Figure 8 illustrates the population of twelve hydrophobic contacts in ten different trajectories. The populations of the former seven pair hydrophobic contacts are very similar among ten trajectories. However, the populations of later five hydrophobic contacts have large fluctuation. If we just sample one simulation, some stable hydrophobic contacts will be missed. Therefore, multiple simulations are necessary for this study. Secondly, we check if ten trajectories are sufficient to sample the conformer space of these systems. A representative population of hydrophobic contact between Leu141 and Tyr650 is listed in supplementary Figure S6. The standard error gradually decreases with the number of simulation, then it keeps constant fluctuation. This is consistent with the previous report that a small number of MD simulations (5–10) are sufficient to capture the average properties of a protein observed in experiment.[48]

### Unfolding and folding pathways

**Figure 9:**
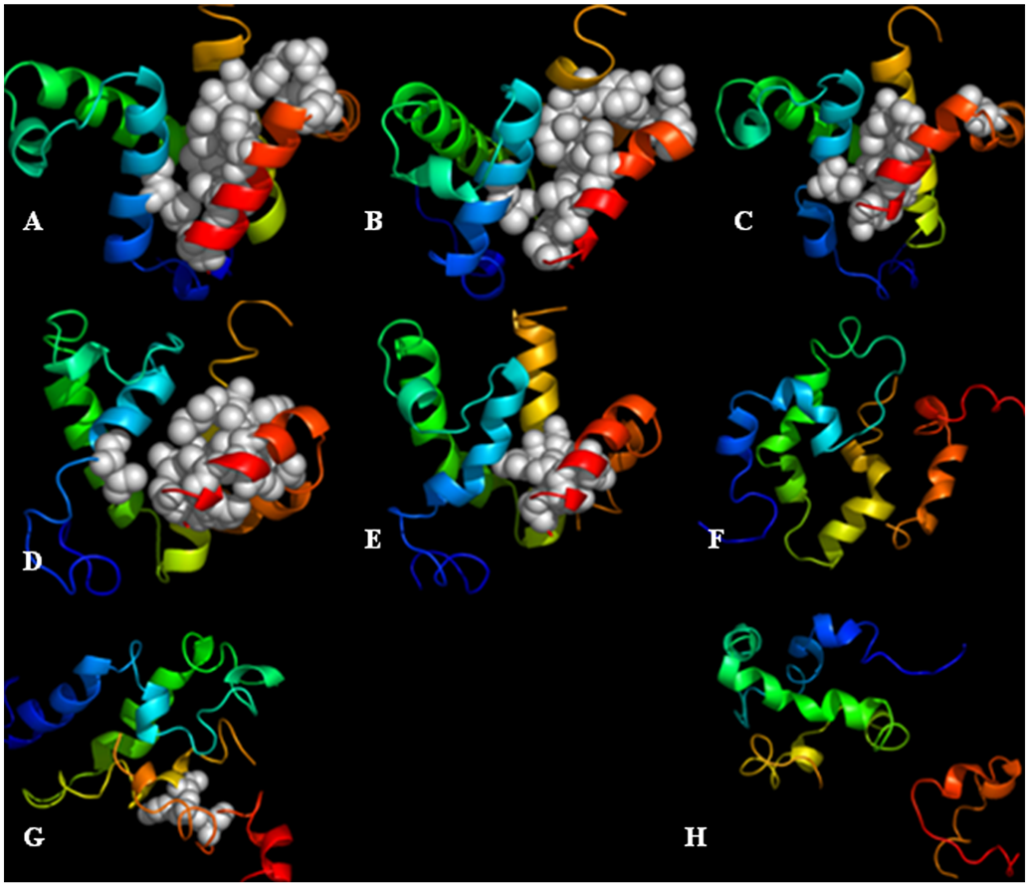
The unfolding pathway of bound pKID. A: <0 ns (F), B:0.17 ns (τQf1), C: 0.35 ns (τQb1), D:1.24 ns (ταA), E: 2.86 ns (ταB), F: 5.98 ns (τQf2), G: 9.44 ns (τQb2) and H:>11 ns (U).

Based on the unfolding kinetics and the landscape analysis, the unfolding pathway for bound pKID can now be constructed and shown in Figure 9. 1) At the first half-time of tertiary unfolding, there are 32 out of 41 (folded state) native hydrophobic contacts within pKID. Most lost hydrophobic contacts are located within the helix α_A_. The native binding contacts between pKID and KIX also start to disappear: only 21 out of 31 exist. There are 62.5% native helical content remaining. 2) At the first half-time of unbinding, there are 32 native hydrophobic contacts within pKID. 54.8% native binding contacts and 79.2% helical content are remained. 3) At the half-time of the helix α_A_ unfolding, there are 28 native hydrophobic contacts within pKID. Four of lost native hydrophobic contacts are also within the helix α_A_. pKID still partly binds KIX. There are 66.7% helical content remaining. 4) At the half-time of the helix α_B_ unfolding, there are 27 native hydrophobic contacts. The helix α_B_ began to unfold. There are 54.8% native binding contacts and 62.5% helical content remaining. 5) At the second half time of tertiary unfolding, there are 46.3% native hydrophobic contacts remaining. pKID begins to move away from the binding site of KIX. 58.3% helical content is remaining. 6) At the second half time of unbinding, there are 53.7% native hydrophobic contacts and one native binding contact remaining.

Because the unfolding pathways of chymotrypsin inhibitor 2 and engrailed homeodomain are confirmed to be the reverse to the folding pathways,[49], [50] we assume that the folding pathway of pKID also obeys the same rule. Therefore, the proposed folding/binding pathway of bound pKID is KIX access, initiation of pKID tertiary folding, folding of the helix α_B_, folding of the helix α_A_, completion of pKID tertiary folding, and finalization of KIX-binding. This suggests that KIX-binding induces the folding of pKID. Our data show that the folding pathway of apo-pKID is different from bound pKID: the folding order of helices α_A_ and α_B_ is reversed. Our results suggest that different folding pathways of bound and apo-pKID determine the different structures and functions of proteins.

### Binding induced-fit mechanism

To date, two main hypotheses are used to explain the folding of ligand binding coupled protein conformational adjustment. [51] One is the “induced-fit” model[52], the other is “conformational selection”.[53], [54], [55], [56], [57], [58] If the bound conformation of the protein exists prior to ligand binding, the ligand will directly select bound conformation, otherwise it will adjust receptor conformation before binding.[27] Recently, residual dipolar coupling is used to identify the folding of ubiquitin complex with conformational selection rather than induced-fit mechanism.[59] Nevertheless, the kinetics character for both mechanisms has been observed in the same system.[60], [61] As for pKID/KIX system, the folding pathway of bound pKID shows that KIX binding is prior to pKID folding. This suggests that bound pKID conformation is formed only after KIX-binding and pKID folding obey an induced-fit mechanism. Furthermore, the average structures of two transition states include native binding contacts. These native contacts are favored to the formation of bound conformation. Finally, because KIX is a relative large ligand with 81 residues, the interaction energy between pKID and KIX is likely to be very large. These strong interactions can stabilize the binding interface between pKID and KIX and favor the induced-fit pathway.[61]

## Supporting Information

Figure S1 Two-dimensional representation for the interaction mode between pKID and KIX, drawn by LIGPLOT program.
(0.42 MB TIF)

Figure S2 Kinetics fitting for apo-pKID
(0.16 MB TIF)

Figure S3 A representative transition probability P for TS1 and TS2 of bound pKID, TS of apo-pKID for snapshot in the transition region for one of trajectories, respectively. The red line is the fit to P = 1/{1+exp[(τ-τTS)/τtrans]}.
(0.14 MB TIF)

Figure S4 Average TSE structures. A: TS1 for bound pKID. B: TS2 for bound pKID. C: TS for apo-pKID.
(0.22 MB TIF)

Figure S5 Intermediate state of bound pKID
(0.19 MB TIF)

Figure S6 The average population of hydrophobic contact vs number of simulations.
(0.02 MB TIF)
