# Supplementary figures and images for: Molecular Dynamics Simulation of Phosphorylated KID Post-Translational Modification
Source: PLoS One. 2009 Aug 5;4(8):e6516. doi: 10.1371/journal.pone.0006516 (PMC2717803; doi:10.1371/journal.pone.0006516)

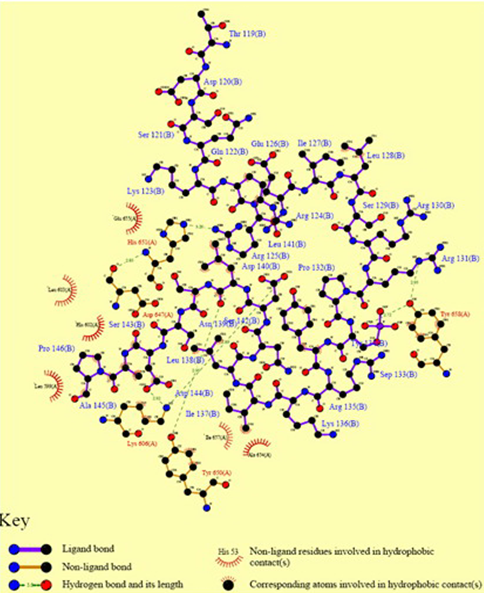

Supplement: Figure S1 — Two-dimensional representation for the interaction mode between pKID and KIX, drawn by LIGPLOT program. (0.42 MB TIF) [file pone.0006516.s001.tif]

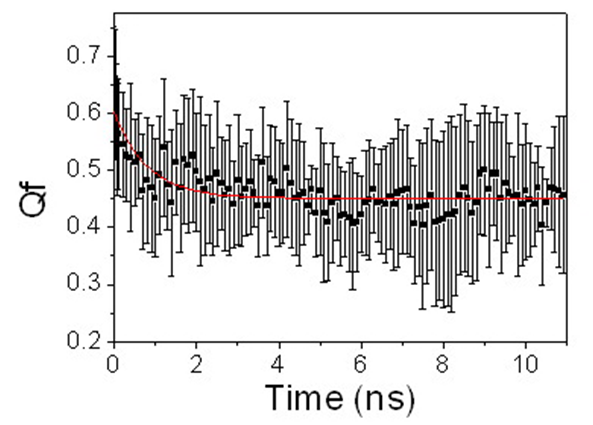

Supplement: Figure S2 — Kinetics fitting for apo-pKID (0.16 MB TIF) [file pone.0006516.s002.tif]

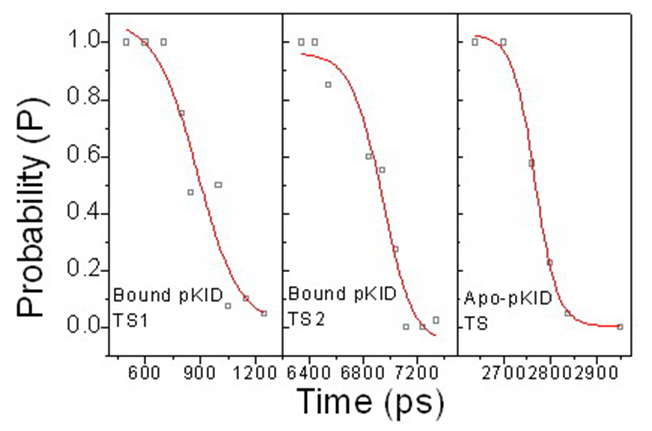

Supplement: Figure S3 — A representative transition probability P for TS1 and TS2 of bound pKID, TS of apo-pKID for snapshot in the transition region for one of trajectories, respectively. The red line is the fit to P = 1/{1+exp[(τ-τTS)/τtrans]}. (0.14 MB TIF) [file pone.0006516.s003.tif]

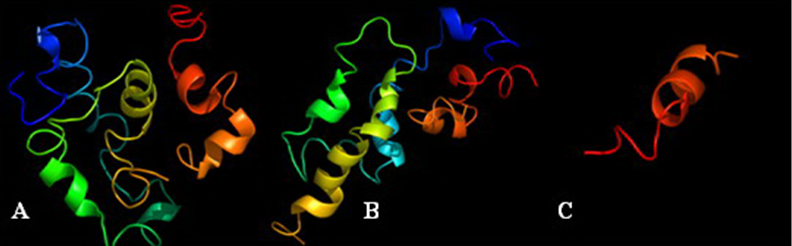

Supplement: Figure S4 — Average TSE structures. A: TS1 for bound pKID. B: TS2 for bound pKID. C: TS for apo-pKID. (0.22 MB TIF) [file pone.0006516.s004.tif]

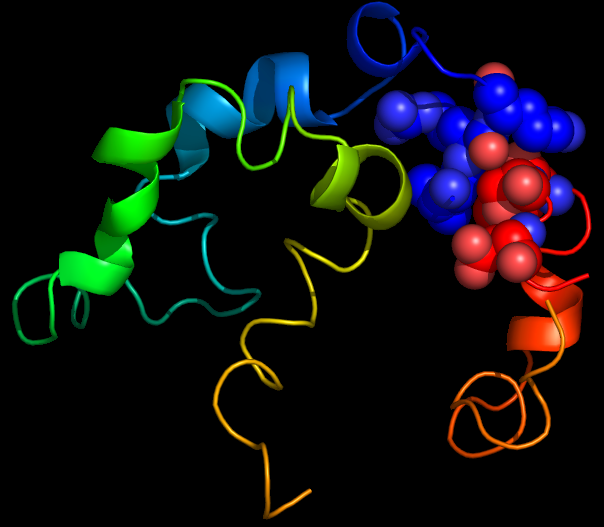

Supplement: Figure S5 — Intermediate state of bound pKID (0.19 MB TIF) [file pone.0006516.s005.tif]

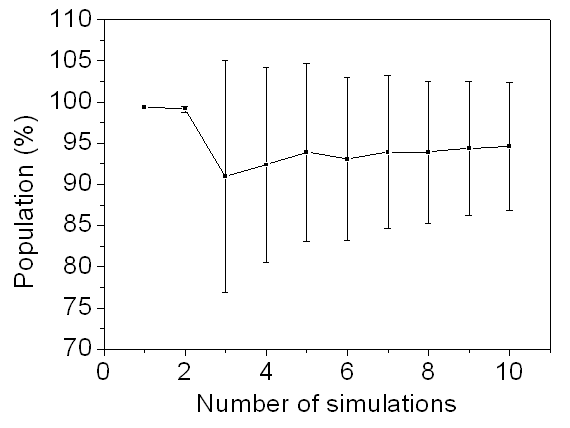

Supplement: Figure S6 — The average population of hydrophobic contact vs number of simulations. (0.02 MB TIF) [file pone.0006516.s006.tif]
